# Supplementary material for: Deubiquitinase USP13 dictates MCL1 stability and sensitivity to BH3 mimetic inhibitors
Source: Nat Commun. 2018 Jan 15;9:215. doi: 10.1038/s41467-017-02693-9 (PMC5768685; doi:10.1038/s41467-017-02693-9)
Supplement: Supplementary file 1 — Supplementary Information [file 41467_2017_2693_MOESM1_ESM.pdf]

Supplementary Figure 1.

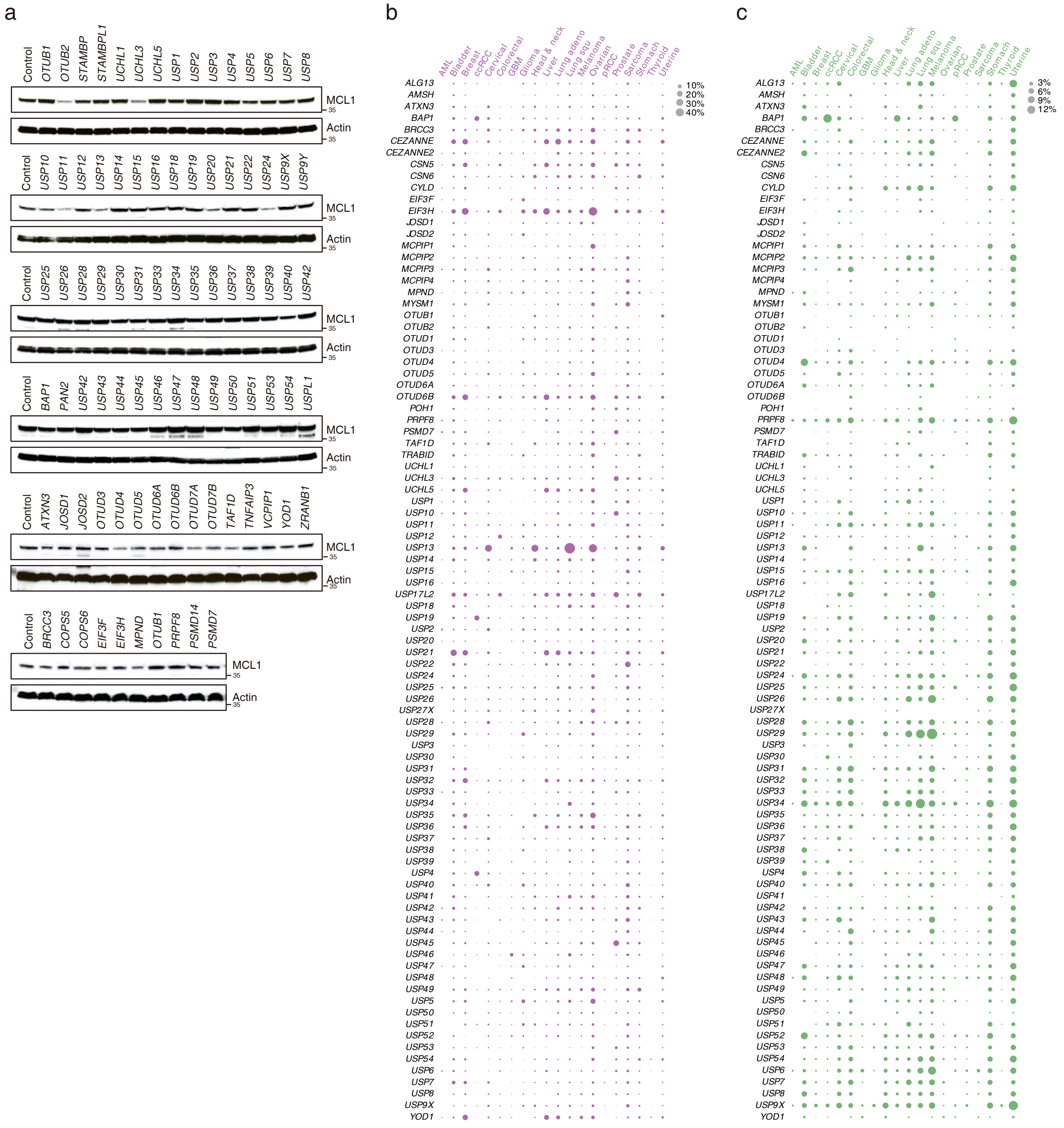

Supplementary Figure 1. (a) The siRNA pools for 84 DUBs were individually transfected into HEK293T cells for 48 hours and the MCL1 protein levels were detected by western blotting. (b) Pan-cancer analysis of copy number alterations of the 84 DUBs genes in cBioportal database. (c) Pan-cancer analysis of somatic mutations of the 84 DUBs genes in cBioportal database.

Supplementary Figure 2.

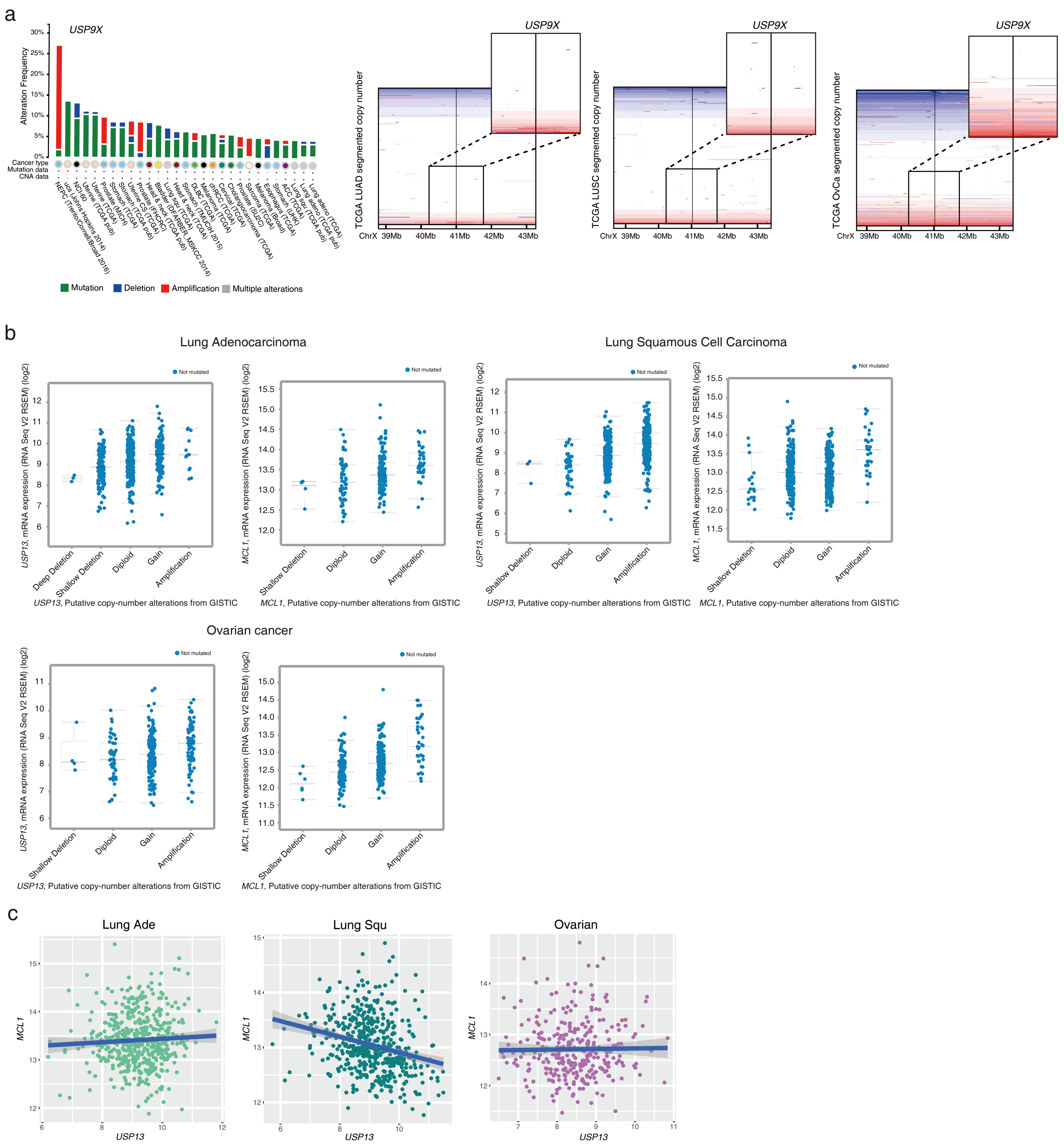

Supplementary Figure 2. (a) Pan-cancer analysis of *USP9X* genomic alterations in cBioportal database. Copy numbers of *USP9X* in TCGA lung cancer and ovarian cancer samples were shown. Color scale: amplification in red and deletion in blue. (b) *USP13* and *MCL1* gene expression in lung and ovarian cancer with different copy numbers. (c) Correlation analysis of *USP13* and *MCL1* gene expression in cBioportal RNAseq database of lung and ovarian cancer.

Supplementary Figure 3.

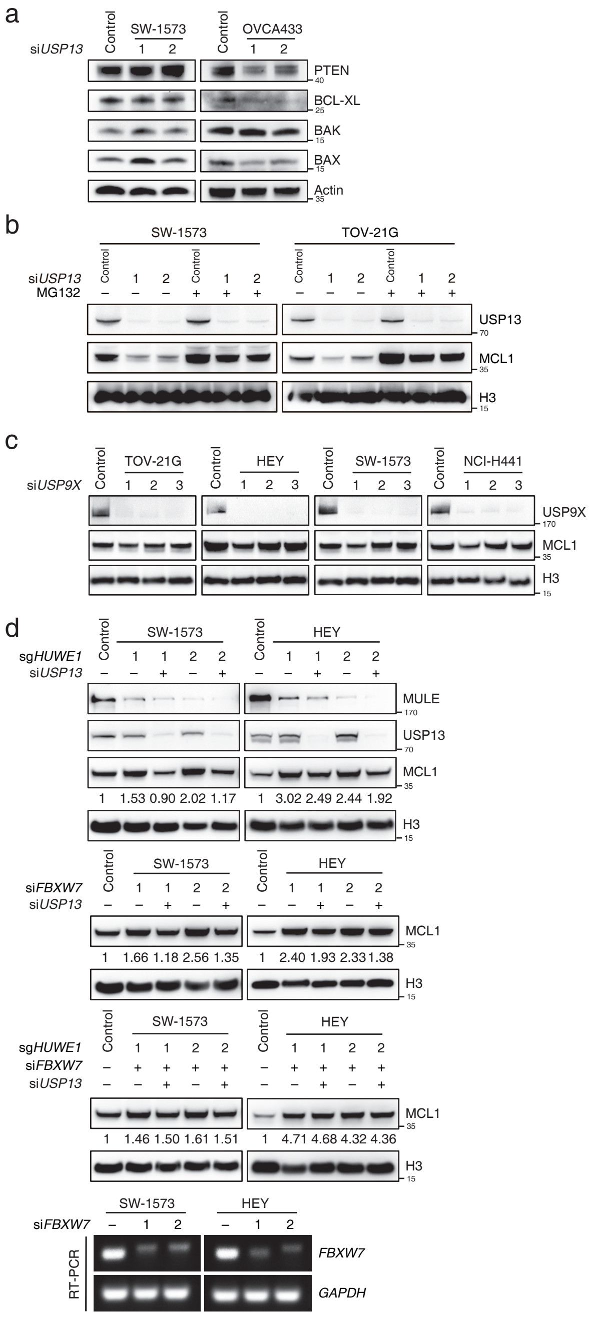

Supplementary Figure 3. (a) SW-1573 and OVCA433 cells were transfected with control or two independent *USP13* siRNAs for 48 hours. The endogenous PTEN, BCL-XL, BAK and BAX protein levels were measured by western blotting. (b) *USP13* was knocked down by siRNAs in SW-1573 and TOV-21G cells with or without the proteasome inhibitor MG132. The endogenous MCL1 protein levels were measured by western blotting. (c) TOV-21G, HEY, SW-1573 and NCI-H441 cells were transfected with control or two independent *USP9X* siRNAs for 48 hours. The endogenous USP9X and MCL1 protein levels were measured by western blotting. (d) *USP13*, *MULE* and *FBXW7* was knocked down in SW-1573 and HEY cells as indicated. The endogenous MCL1 protein levels were measured by western blotting.

Supplementary Figure 4.

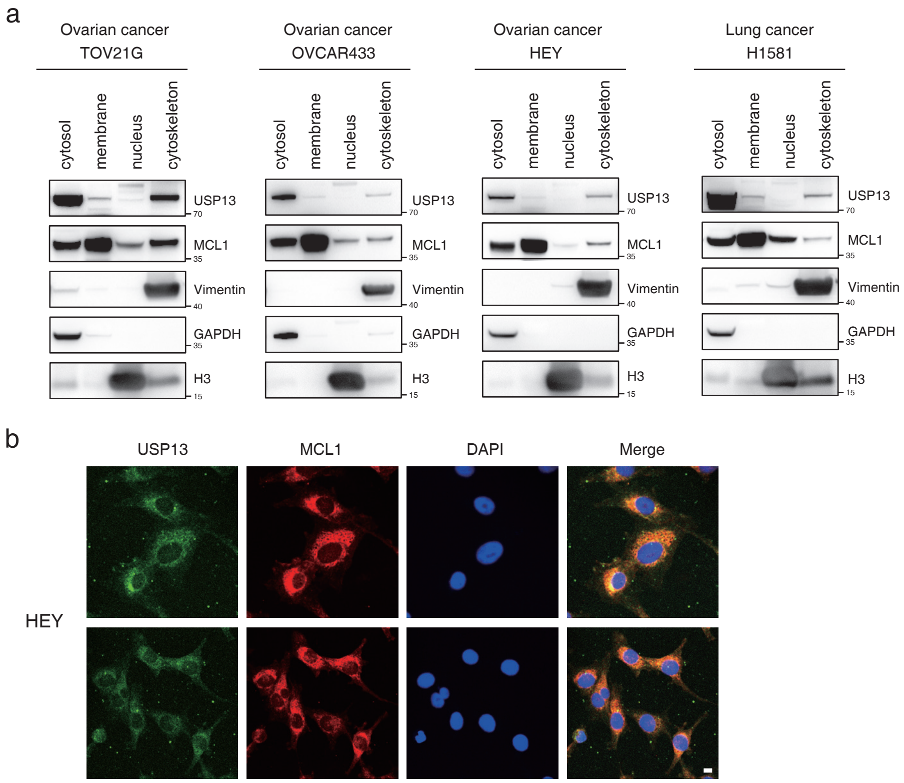

Supplementary Figure 4. (a) Western blot analysis of indicated proteins in cytosol, mitochondria-enriched membrane, nucleus, and cytoskeleton fractions prepared from TOV-21G, OVCA433, HEY and NCI-H1581 cells. (b) Immunofluorescent staining of USP13 (green) and MCL1 (red) in HEY cells. The right panels were the overlay of USP13, MCL1 and nuclear 4',6-diamidino-2-phenylindole (DAPI; blue) staining of the same field. Scale bar, 10 μm.

Supplementary Figure 5.

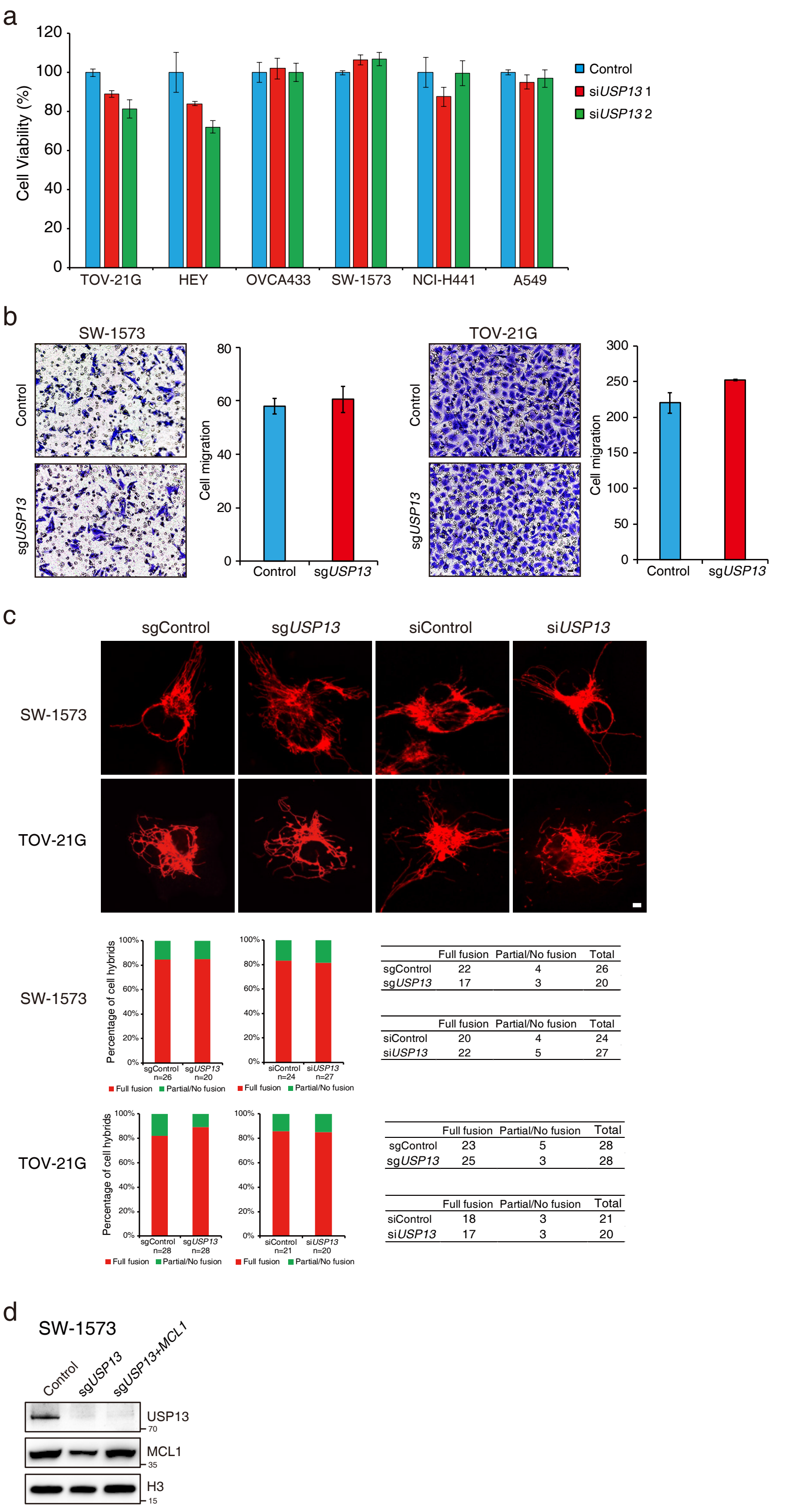

SW-1573:

	Full fusion	Partial/No fusion	Total
sgControl	22	4	26
sgUSP13	17	3	20

	Full fusion	Partial/No fusion	Total
siControl	20	4	24
siUSP13	22	5	27

TOV-21G:

	Full fusion	Partial/No fusion	Total
sgControl	23	5	28
sgUSP13	25	3	28

	Full fusion	Partial/No fusion	Total
siControl	18	3	21
siUSP13	17	3	20

Supplementary Figure 5. (a) Cell viability of TOV-21G, HEY, OVCA433, SW-1573, NCI-H441 and A549 with or without *USP13* knockdown. Error bars indicated standard deviation (each condition contained three biological replicates). (b) Transwell migration assay of SW-1573 and TOV-21G cells with or without *USP13* depletion. Image magnification, 200×. Quantification was based on the average number of migrated cells in 3 fields and error bars indicated standard deviation. (c) Mitochondrial morphology analysis in SW-1573 and TOV-21G cells upon *USP13* knockout or knockdown. (d) Western blotting for the overexpression of *MCL1* in *USP13*-depeleted SW-1573 cells. Scale bar, 10 μm.

Supplementary Figure 6.

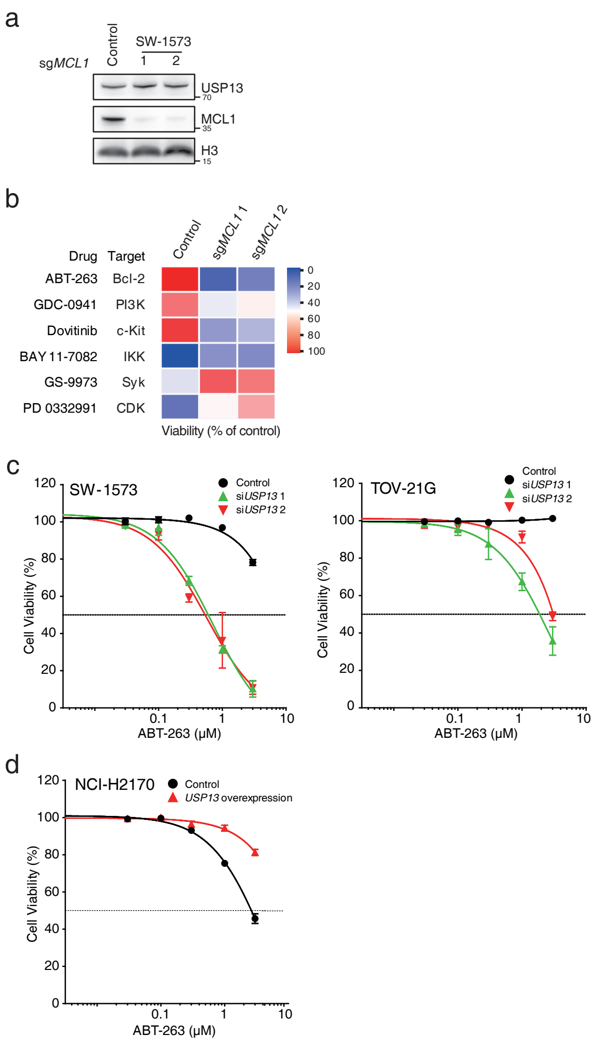

Supplementary Figure 6. (a) *MCL1* was knocked out in SW-1573 cells using CRISPR/Cas9. (b) The resultant *MCL1*-depleted cells were screened on a collection of 180 small molecule inhibitors targeting different signaling pathways. Cell viability in triplicate wells was determined using Cell Counting Kit-8. The heatmap showed compounds with different sensitivity upon *MCL1* depletion. (c) *USP13* was knocked down in SW-1573 and TOV-21G cells using siRNAs, cells were treated with ABT-263 for 96 hours and cell viability was detected by Cell Counting Kit-8. Error bars indicated standard deviation (each condition contained three biological replicates). (d) *USP13* was overexpressed in NCI-H2170 cells using siRNAs, cells were treated with ABT-263 for 96 hours and cell viability was detected by Cell Counting Kit-8. Error bars indicated standard deviation (each condition contained three biological replicates).

Supplementary Figure 7.

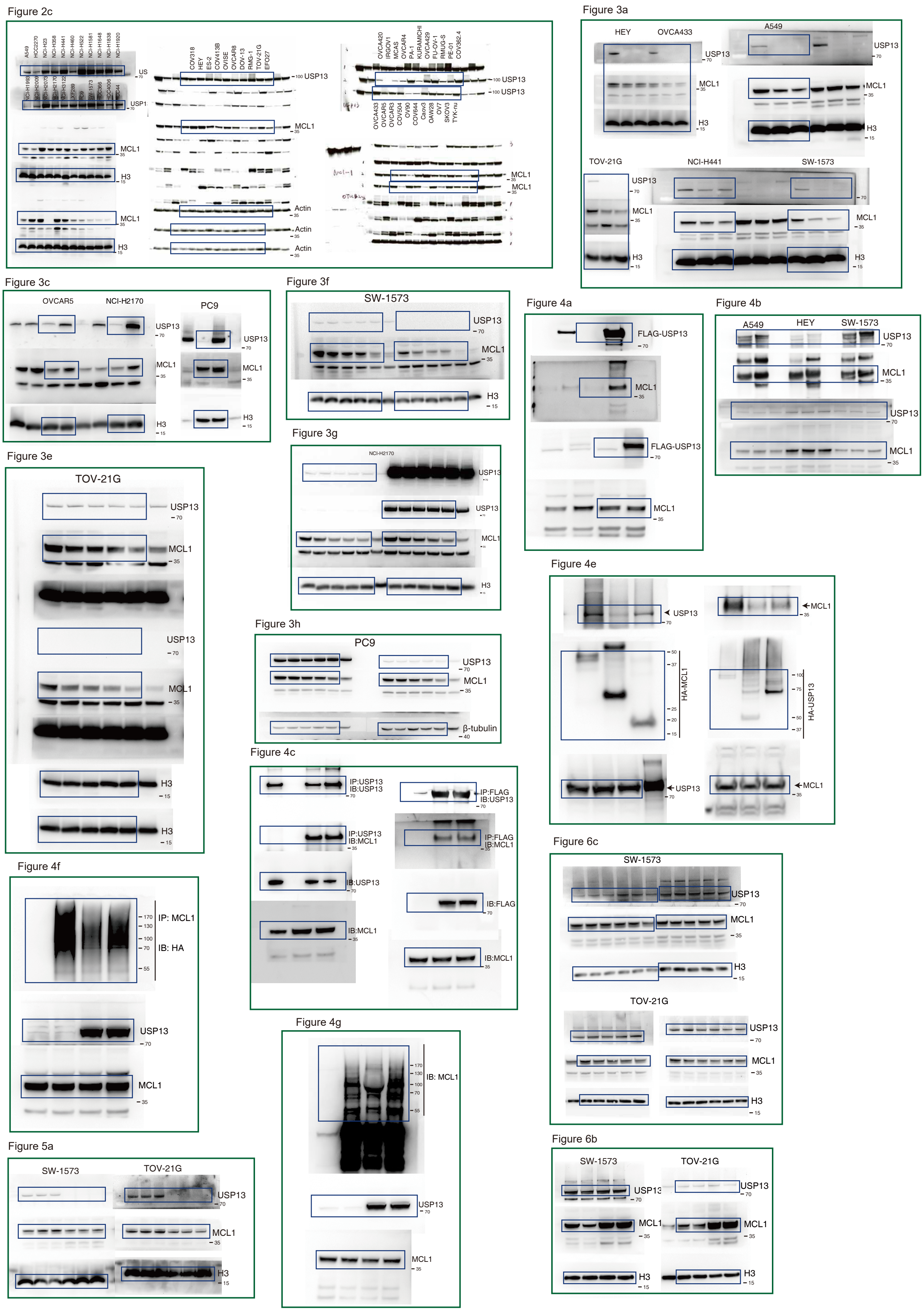

Supplementary Figure 7. All uncropped blots and gels data for Figures.

Supplementary Figure 8.

Supplementary Figure 1a

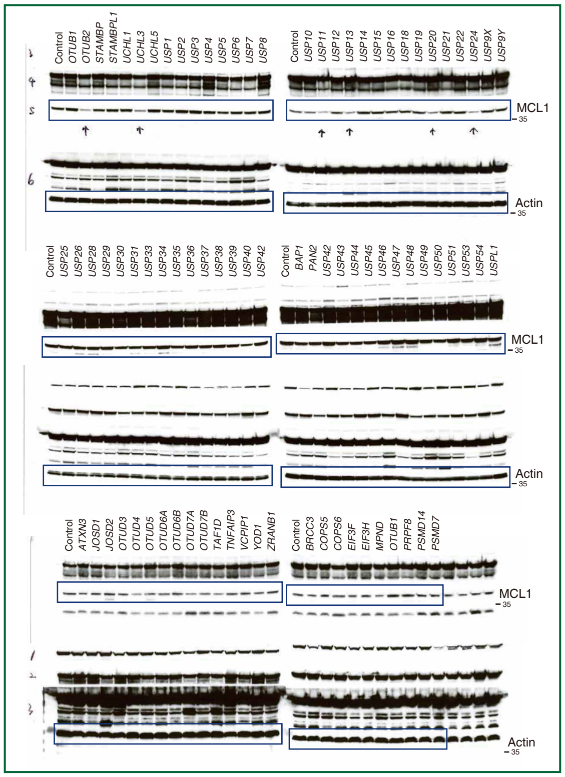

Supplementary Figure 3a

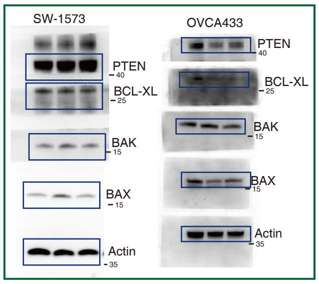

Supplementary Figure 3b

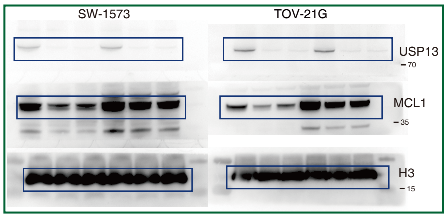

Supplementary Figure 3c

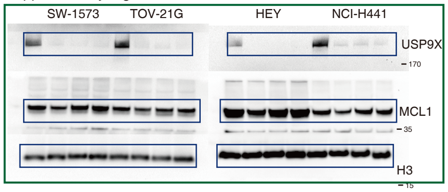

Supplementary Figure 3d

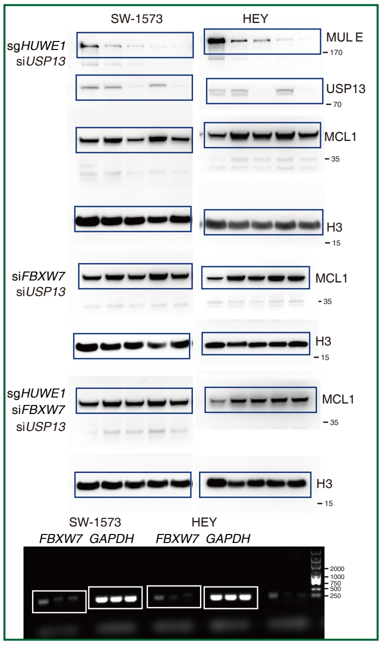

Supplementary Figure 4a

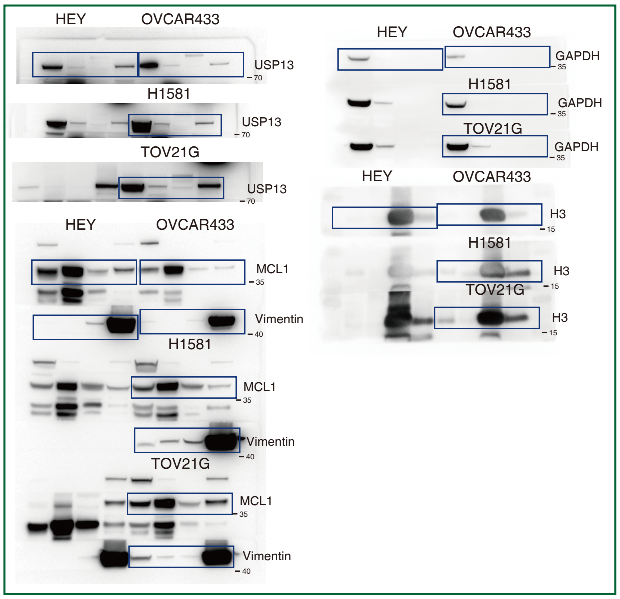

Supplementary Figure 5d

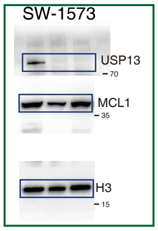

Supplementary Figure 6a

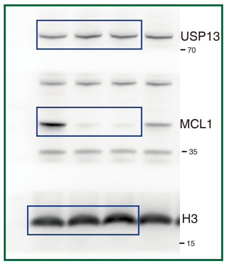

Supplementary Figure 8. All uncropped blots and gels data for Supplementary Figures.

Supplementary Table 1: TCGA pan-cancer genomic interrogation of copy number alterations in the 84 DUBs

STUDY_ABBREVIATION	AML (TCGA)	Bladder (TCGA)	Breast (TCGA)	ccRCC (TCGA)	Cervical (TCGA)	Colorectal (TCGA)	GBM (TCGA)	Glioma (TCGA)	Head & neck (TCGA)	Liver (TCGA)	Lung adeno (TCGA)	Lung squ (TCGA)	Melanoma (TCGA)	Ovarian (TCGA)	pRCC (TCGA)	Prostate (TCGA)	Sarcoma (TCGA)	Stomach (TCGA)	Thyroid (TCGA)	Uterine (TCGA)
samples	200	413	1105	538	309	633	604	530	530	442	522	504	479	603	293	499	265	478	516	548
UCHL1	0.00%	0.70%	1.30%	0.40%	0.30%	0.20%	0.20%	0.40%	0.40%	0.50%	1.00%	1.20%	0.80%	2.60%	0.00%	1.80%	0.00%	1.10%	0.00%	1.30%
UCHL3	0.50%	4.20%	1.70%	0.60%	2.00%	1.90%	1.20%	1.20%	2.30%	2.20%	1.90%	1.60%	0.50%	2.40%	0.00%	9.80%	3.10%	3.40%	1.00%	0.90%
BAP1	0.00%	3.40%	0.70%	10.20%	3.10%	0.50%	0.20%	1.80%	1.00%	1.10%	0.40%	1.00%	0.30%	2.40%	0.30%	1.00%	1.90%	1.80%	0.00%	2.80%
UCHL5	0.50%	3.70%	9.80%	0.20%	1.40%	0.80%	0.00%	0.00%	1.00%	10.30%	5.80%	1.80%	4.40%	7.10%	0.00%	1.60%	1.90%	1.40%	0.60%	3.00%
USP1	0.50%	1.20%	1.50%	0.00%	1.40%	0.00%	0.30%	0.20%	0.40%	0.50%	1.70%	0.80%	0.80%	4.70%	0.00%	2.40%	4.70%	0.50%	0.00%	1.10%
USP2	1.60%	1.20%	1.00%	0.40%	4.40%	0.20%	0.70%	2.50%	1.50%	0.50%	2.30%	1.00%	3.50%	2.90%	1.00%	2.00%	3.50%	1.60%	0.00%	1.30%
USP3	0.00%	1.00%	0.80%	0.00%	0.30%	0.00%	0.20%	0.40%	0.00%	1.10%	1.40%	0.20%	0.50%	2.80%	0.00%	0.40%	1.60%	0.90%	0.00%	1.50%
USP4	0.00%	2.50%	0.70%	10.60%	1.70%	0.50%	0.20%	1.40%	0.80%	1.40%	0.20%	1.00%	0.30%	2.10%	0.00%	0.80%	2.30%	1.80%	0.00%	1.50%
USP5	0.00%	2.00%	3.40%	0.00%	0.70%	0.60%	1.40%	6.00%	1.90%	0.50%	2.70%	4.20%	1.10%	11.20%	0.30%	0.80%	1.60%	1.60%	0.00%	2.60%
USP6	1.00%	2.20%	0.90%	0.00%	0.30%	1.00%	0.20%	0.20%	1.00%	2.20%	1.40%	0.40%	1.10%	1.20%	0.30%	2.40%	2.70%	0.90%	0.20%	0.70%
USP7	0.00%	5.60%	4.70%	0.20%	2.40%	0.00%	0.20%	0.40%	0.80%	1.10%	1.20%	1.20%	0.50%	2.60%	0.70%	1.00%	3.10%	1.60%	0.00%	0.70%
USP8	0.00%	1.00%	1.50%	0.00%	1.00%	0.60%	0.30%	0.80%	0.40%	0.00%	1.60%	0.80%	1.60%	2.10%	0.00%	1.20%	1.60%	0.50%	0.00%	0.60%
USP9X	0.00%	0.70%	0.70%	0.40%	2.00%	0.60%	0.30%	1.80%	2.50%	1.10%	1.70%	2.00%	0.50%	3.10%	0.30%	0.20%	4.70%	1.60%	0.20%	0.70%
USP10	1.00%	2.20%	2.90%	0.40%	1.00%	0.30%	0.20%	0.00%	0.20%	0.50%	2.50%	0.20%	0.80%	2.90%	1.40%	9.60%	1.60%	2.00%	0.60%	1.70%
USP11	0.00%	1.20%	1.90%	0.60%	2.00%	0.80%	0.30%	2.10%	2.30%	0.80%	1.90%	2.60%	0.50%	6.20%	0.30%	0.60%	4.30%	1.60%	0.20%	2.60%
USP12	0.00%	2.00%	2.00%	0.40%	0.70%	6.80%	0.50%	2.30%	0.80%	0.50%	1.90%	0.60%	0.30%	1.20%	0.00%	1.20%	3.50%	2.70%	0.60%	0.20%
USP13	0.00%	4.70%	4.60%	1.70%	20.00%	0.20%	2.80%	1.20%	20.50%	2.20%	2.90%	45.90%	1.10%	28.00%	0.70%	3.30%	1.60%	6.10%	0.00%	8.20%
USP14	0.50%	4.40%	1.40%	0.00%	2.40%	0.60%	1.00%	0.80%	5.00%	1.40%	2.70%	4.60%	1.40%	4.00%	0.00%	0.80%	3.50%	1.40%	0.20%	2.20%
USP15	0.00%	0.70%	1.30%	0.00%	0.70%	0.20%	1.90%	1.00%	1.30%	0.50%	2.70%	1.20%	0.50%	2.20%	0.00%	0.60%	5.80%	2.50%	0.00%	0.40%
USP16	0.50%	1.50%	1.10%	0.20%	0.70%	0.20%	0.00%	0.60%	0.20%	0.30%	1.60%	1.20%	0.30%	2.10%	0.00%	0.80%	2.30%	0.90%	0.00%	0.40%
USP17L2	0.00%	7.60%	6.30%	1.10%	2.00%	6.30%	0.30%	0.40%	3.60%	7.60%	6.40%	6.20%	2.70%	8.50%	0.70%	12.00%	1.90%	7.70%	0.00%	4.50%
USP18	0.00%	3.70%	1.30%	0.00%	1.00%	0.20%	0.70%	0.60%	2.70%	0.80%	2.50%	6.00%	2.50%	4.70%	0.30%	1.00%	5.10%	1.60%	0.40%	1.50%
USP19	0.00%	2.70%	0.80%	10.60%	1.70%	0.50%	0.20%	1.60%	0.80%	1.10%	0.20%	0.80%	0.30%	2.10%	0.00%	0.80%	1.90%	1.80%	0.00%	0.70%
USP20	0.50%	0.70%	0.80%	0.20%	0.30%	0.00%	0.50%	1.00%	1.70%	0.80%	0.80%	0.60%	0.30%	2.10%	0.30%	1.60%	0.80%	2.30%	0.80%	1.50%
USP21	0.00%	16.90%	12.60%	0.60%	3.10%	0.60%	0.00%	0.60%	1.50%	12.20%	10.30%	5.60%	3.80%	4.70%	0.30%	1.40%	6.20%	2.50%	0.20%	4.60%
USP22	0.00%	4.20%	2.60%	0.00%	1.00%	1.10%	0.70%	0.40%	1.10%	3.00%	2.10%	3.40%	0.80%	2.20%	0.00%	1.60%	13.20%	1.80%	0.60%	1.70%
USP24	0.00%	2.70%	1.40%	0.00%	1.00%	0.00%	0.20%	0.40%	0.20%	0.30%	1.70%	0.60%	0.30%	6.20%	0.00%	2.00%	3.90%	0.20%	0.00%	1.30%
USP25	1.60%	2.90%	2.50%	0.20%	1.70%	1.10%	0.90%	1.00%	2.10%	0.30%	3.10%	3.60%	1.40%	4.80%	0.00%	1.20%	5.10%	2.30%	0.00%	0.70%
USP26	0.00%	1.20%	1.10%	0.20%	1.70%	0.60%	0.30%	1.40%	1.30%	1.40%	1.70%	1.00%	0.30%	2.60%	0.30%	0.60%	2.70%	1.40%	0.40%	0.40%
USP27X	0.00%	1.20%	1.20%	0.60%	2.40%	0.80%	0.30%	1.90%	2.30%	0.50%	1.90%	2.20%	0.50%	7.10%	0.30%	0.00%	3.10%	1.60%	0.20%	2.00%
USP28	1.00%	1.00%	1.40%	0.40%	4.70%	0.30%	0.50%	0.80%	1.50%	0.30%	1.90%	1.20%	4.10%	4.00%	1.70%	4.10%	3.90%	1.60%	0.00%	1.10%
USP29	0.00%	3.90%	2.60%	0.40%	1.40%	0.20%	0.50%	5.70%	1.00%	0.80%	0.80%	2.80%	0.30%	4.10%	0.30%	0.60%	1.20%	2.00%	0.00%	1.90%
USP30	0.00%	1.50%	0.30%	0.00%	0.00%	0.00%	0.50%	1.60%	0.40%	0.50%	0.80%	0.20%	0.80%	2.20%	0.30%	1.60%	2.30%	0.20%	0.00%	0.40%
USP31	0.00%	2.20%	4.10%	0.20%	0.00%	0.50%	0.30%	0.20%	0.20%	0.50%	0.60%	0.00%	0.30%	1.20%	0.30%	0.60%	0.00%	0.20%	0.00%	0.60%
USP32	0.00%	4.20%	11.00%	0.60%	1.70%	1.10%	0.50%	2.10%	0.20%	5.90%	3.70%	2.20%	4.40%	4.00%	1.40%	1.20%	2.70%	2.90%	0.60%	2.40%
USP33	1.00%	2.20%	1.40%	0.00%	1.00%	0.50%	0.20%	0.40%	0.20%	0.80%	1.70%	1.00%	0.50%	2.60%	0.00%	2.80%	2.70%	0.70%	0.00%	0.70%
USP34	0.00%	2.90%	1.30%	0.40%	0.30%	0.00%	0.20%	0.00%	0.80%	0.50%	1.00%	7.00%	0.50%	3.50%	0.30%	0.80%	0.40%	1.10%	0.00%	0.90%
USP35	0.00%	3.70%	7.60%	0.20%	2.00%	0.30%	0.90%	0.00%	4.80%	1.10%	4.10%	1.80%	4.40%	13.00%	0.30%	0.80%	3.10%	3.60%	0.00%	2.20%
USP36	0.00%	2.20%	5.90%	0.60%	1.70%	0.50%	0.00%	2.10%	0.20%	5.90%	3.50%	3.00%	5.20%	6.60%	1.00%	1.20%	4.30%	1.60%	0.60%	1.90%
USP37	0.00%	2.00%	1.10%	0.90%	3.70%	0.20%	0.20%	0.60%	1.50%	0.80%	0.40%	1.40%	0.30%	2.40%	1.40%	1.20%	3.10%	1.10%	0.20%	1.70%
USP38	0.00%	2.00%	0.70%	0.80%	1.00%	0.20%	0.20%	2.30%	0.20%	0.50%	0.60%	2.20%	0.80%	2.60%	0.00%	0.40%	1.90%	1.10%	0.00%	0.70%
USP39	0.00%	0.00%	3.90%	20.70%	0.00%	1.30%	1.80%	0.90%	0.00%	0.00%	0.00%	0.00%	0.00%	0.00%	0.00%	0.00%	0.40%	0.00%	1.10%	0.00%
USP40	0.00%	2.90%	0.60%	1.30%	3.70%	0.20%	0.30%	4.30%	1.50%	0.50%	1.00%	0.80%	0.80%	2.60%	1.70%	0.60%	7.00%	0.70%	0.20%	1.10%
USP41	0.00%	3.70%	1.30%	0.00%	1.00%	0.20%	0.70%	0.60%	2.70%	0.80%	2.50%	6.00%	2.50%	4.70%	0.30%	1.00%	5.10%	1.60%	0.40%	1.50%
USP42	1.00%	4.20%	1.10%	0.20%	0.70%	0.20%	0.50%	1.40%	2.10%	0.80%	4.30%	1.00%	3.30%	3.10%	0.70%	0.80%	4.70%	3.40%	0.00%	0.70%
USP43	1.00%	2.50%	1.20%	0.20%	0.00%	1.10%	0.30%	0.40%	0.60%	2.20%	2.30%	1.00%	0.80%	2.20%	0.30%	3.00%	7.00%	1.10%	0.20%	1.10%
USP44	0.50%	1.20%	0.80%	0.00%	0.30%	0.00%	0.30%	0.80%	0.20%	0.50%	0.40%	0.00%	0.50%	1.20%	0.00%	1.60%	3.90%	0.70%	0.00%	0.70%
USP45	0.00%	1.70%	1.80%	0.20%	1.40%	0.60%	0.30%	0.00%	0.80%	1.90%	1.00%	0.60%	3.00%	2.60%	0.00%	13.80%	2.70%	2.70%	0.00%	0.70%
USP46	0.00%	0.50%	0.60%	0.40%	0.70%	0.30%	5.40%	2.10%	1.10%	0.00%	1.60%	5.00%	1.60%	1.20%	0.00%	1.00%	3.10%	1.40%	0.00%	0.60%
USP47	0.50%	1.00%	1.20%	0.00%	0.00%	0.20%	0.50%	3.70%	0.40%	0.50%	1.60%	1.20%	0.00%	1.70%	0.00%	0.40%	1.20%	0.00%	0.00%	1.30%
USP48	0.00%	0.50%	4.70%	10.30%	1.00%	1.20%	0.90%	1.90%	0.00%	0.00%	0.00%	1.40%	0.00%	0.00%	1.10%	0.00%	0.40%	0.00%	3.60%	2.90%
USP49	0.00%	2.00%	2.80%	0.40%	1.00%	1.10%	0.50%	0.80%	1.30%	3.20%	3.50%	2.60%	3.80%	6.60%	1.00%	0.60%	4.70%	6.30%	0.00%	1.30%
USP50	0.00%	1.00%	1.50%	0.00%	1.00%	0.60%	0.30%	0.80%	0.40%	0.00%	1.60%	0.80%	1.60%	1.90%	0.00%	1.20%	1.20%	0.90%	0.00%	0.60%
USP51	0.00%	0.00%	1.60%	0.80%	1.40%	0.50%	0.50%	3.30%	2.30%	1.10%	1.20%	1.80%	1.10%	2.80%	0.30%	0.20%	3.50%	1.10%	0.00%	0.90%
USP52	0.00%	1.50%	0.20%	0.00%	1.00%	0.30%	1.60%	1.40%	0.20%	1.40%	2.10%	0.40%	0.80%	3.30%	0.00%	0.40%	3.50%	1.40%	0.00%	1.30%
USP53	0.00%	1.00%	0.50%	0.40%	0.70%	0.30%	0.20%	1.00%	0.20%	0.30%	0.00%	0.40%	0.30%	3.10%	0.00%	3.50%	2.30%	0.90%	0.00%	0.70%
USP54	0.00%	2.50%	2.10%	0.00%	0.30%	0.80%	0.50%	0.20%	0.80%	1.40%	0.60%	1.40%	0.00%	4.30%	0.00%	1.80%	1.60%	3.20%	0.80%	2.40%
CYLD	0.50%	1.00%	2.70%	0.00%	0.70%	0.80%	0.20%	0.00%	1.10%	0.80%	1.90%	2.00%	1.40%	1.00%	0.00%	2.80%	1.90%	0.70%	0.00%	1.10%
CEZANNE	0.00%	9.30%	12.70%	0.60%	3.10%	0.60%	0.20%	0.40%	2.10%	11.10%	13.80%	6.00%	3.00%	10.20%	0.00%	1.40%	7.00%	3.20%	0.00%	6.50%
CEZANNE2	0.50%	1.50%	1.90%	0.20%	0.30%	1.30%	0.70%	1.20%	0.40%	0.30%	3.50%	1.80%	1.60%	3.10%	1.00%	2.20%	1.90%	0.90%	0.00%	0.90%
TRABID	0.00%	2.90%	1.50%	0.20%	0.30%	0.20%	0.70%	2.70%	0.60%	0.30%	0.60%	0.80%	1.10%	6.00%	0.00%	1.40%	0.80%	3.60%	0.00%	1.30%
OTUD4	0.00%	2.00%	0.60%	0.40%	1.00%	0.20%	0.20%	2.30%	0.20%	0.50%	0.40%	1.80%	0.80%	2.40%	0.00%	0.40%	2.70%	0.90%	0.00%	0.70%
ALG13	0.00%	1.20%	0.60%	0.60%	0.30%	0.20%	0.00%	1.40%	1.00%	0.30%	1.00%	1.00%	0.50%	1.90%	0.00%	0.40%	2.30%	0.70%	0.20%	0.40%
OTUD3	0.50%	0.70%	0.70%	0.20%	0.70%	0.30%	0.30%	0.20%	0.20%	0.80%	1.00%	0.40%	1.40%	2.60%	0.30%	0.80%	1.90%	0.90%	0.00%	1.10%
OTUD1	0.00%	2.50%	1.90%	0.00%	0.30%	0.00%	0.20%	1.80%	1.50%	1.10%	0.80%	1.60%	0.50%	4.10%	0.30%	0.40%	2.30%	0.70%	0.00%	1.10%
OTUD6A	0.00%	1.70%	0.60%	0.60%	0.30%	0.50%	0.30%	1.40%	1.00%	0.00%	0.60%	2.00%	0.80%	1.90%	0.00%	0.80%	3.50%	0.50%	0.40%	0.70%
OTUD2/YOD1	0.50%	1.50%	12.40%	0.20%	1.40%	0.80%	0.50%	0.00%	0.80%	10.30%	6.60%	2.00%	4.60%	5.40%	0.00%	0.40%	1.60%	2.30%	0.80%	2.40%
OTUD6B	0.00%	6.40%	13.90%	0.60%	1.40%	4.20%	0.50%	2.50%	4.20%	13.80%	4.30%	3.60%	4.10%	10.90%	0.00%	5.90%	3.10%	4.50%	0.00%	3.90%
OTUB1	0.00%	1.20%	1.00%	0.00%	0.30%	0.20%	0.20%	0.00%	2.50%	0.50%	0.60%	0.20%	0.50%	2.10%	0.00%	0.60%	0.00%	0.50%	0.00%	4.10%
OTUB2	0.00%	2.00%	0.90%	0.20%	3.10%	0.30%	0.50%	1.20%	1.90%	0.80%	1.20%	2.40%	0.50%	2.20%	0.30%	2.60%	1.90%	0.20%	0.00%	0.70%
OTUD5	0.00%	1.00%	1.30%	0.60%	2.40%	0.60%	0.50%	1.90%	1.90%	0.50%	1.90%	2.20%	0.50%	7.40%	0.30%	0.60%	3.10%	1.80%	0.20%	2.40%
ATXN3	0.00%	2.50%	1.20%	0.20%	3.10%	0.30%	0.30%	1.20%	2.10%	0.50%	1.00%	2.00%	0.50%	2.10%	0.70%	2.00%	1.60%	0.00%	0.00%	0.70%
JOSD1	0.00%	2.00%	0.50%	0.00%	0.30%	0.00%	0.20%	1.40%	0.20%	1.10%	0.80%	1.40%	3.50%	2.40%	0.00%	0.00%	0.40%	0.70%	0.00%	1.50%
JOSD2	0.00%	1.70%	1.80%	0.20%	1.40%	0.00%	0.70%	3.70%	0.20%	0.80%	0.20%	0.80%	0.00%	0.90%	0.30%	0.80%	0.00%	0.90%	0.00%	0.90%
TAF1D	0.00%	2.00%	1.80%	0.60%	3.70%	0.30%	0.90%	0.00%	3.30%	0.50%	1.90%	1.40%	3.00%	6.40%	1.00%	0.80%	2.70%	1.10%	0.00%	1.30%
MCPIP1	0.00%	2.20%	1.20%	0.00%	0.70%	0.30%	0.30%	0.20%	0.80%	0.50%	1.00%	0.80%	0.50%	10.40%	0.30%	0.40%	1.20%	1.40%	0.00%	1.90%
MCPIP2	0.00%	1.00%	1.00%	0.80%	0.70%	0.60%	0.30%	2.30%	0.80%	0.00%	0.60%	1.80%	0.50%	2.20%	0.00%	1.00%	4.70%	0.70%	0.20%	0.70%
MCPIP3	1.00%	1.70%	1.90%	0.40%	4.40%	0.60%	0.70%	0.40%	1.90%	0.30%	2.10%	1.20%	4.10%	4.80%	1.70%	1.80%	3.50%	0.90%	0.00%	1.10%
MCPIP4	0.00%	1.50%	1.60%	0.20%	2.40%	0.60%	0.30%	1.80%	0.60%	1.90%	1.40%	0.40%	1.90%	2.10%	0.00%	0.60%	6.20%	1.60%	0.00%	0.60%
POH1	0.00%	2.00%	0.20%	0.20%	0.70%	0.30%	0.30%	0.20%	0.60%	1.90%	0.60%	1.40%	0.00%	3.80%	0.70%	3.90%	2.30%	0.70%	0.00%	0.90%
CSN5	0.50%	3.70%	8.70%	0.80%	1.00%	3.10%	0.20%	0.20%	3.40%	8.90%	3.10%	3.00%	2.70%	8.50%	0.00%	5.90%	4.30%	3.20%	0.00%	2.80%
CSN6	0.00%	1.50%	1.30%	0.60%	1.40%	0.50%	1.20%	2.30%	5.20%	1.60%	3.70%	4.80%	1.60%	5.40%	1.40%	0.80%	1.20%	6.30%	0.20%	2.60%
AMSH	0.00%	1.70%	0.60%	0.00%	0.00%	0.00%	0.00%	0.20%	0.60%	0.00%	0.60%	2.00%	0.30%	2.20%	0.30%	1.40%	0.80%	0.20%	0.00%	1.10%
BRCC3	0.00%	3.00%	1.90%	1.10%	4.00%	1.00%	1.00%	2.70%	3.40%	2.20%	5.00%	3.80%	1.60%	8.60%	0.70%	0.80%	4.70%	4.50%	0.80%	2.00%
MYSM1	0.00%	1.70%	2.00%	0.00%	2.00%	0.30%	0.30%	0.20%	0.60%	0.50%	1.90%	0.60%	0.50%	5.70%	0.00%	2.40%	8.60%	0.50%	0.00%	0.70%
MPND	0.50%	0.20%	1.30%	0.40%	2.40%	0.50%	1.40%	2.50%	0.40%	1.40%	1.00%	0.80%	0.80%	3.30%	0.00%	0.80%	5.10%	2.30%	0.40%	0.70%
PSMD7	1.60%	2.20%	2.20%	0.00%	1.00%	0.30%	0.00%	0.60%	0.00%	0.50%	2.10%	0.20%	1.10%	3.10%	0.30%	7.90%	0.40%	1.10%	0.40%	2.00%
PRPF8	1.00%	2.70%	0.70%	0.20%	0.70%	1.10%	0.30%	0.40%	0.60%	2.70%	1.70%	0.40%	1.10%	2.40%	0.30%	1.20%	2.30%	1.60%	0.20%	1.70%
EIF3H	0.50%	8.10%	19.40%	0.80%	2.40%	4.90%	0.30%	6.80%	8.00%	18.10%	5.80%	5.60%	5.40%	29.70%	0.00%	6.30%	5.10%	6.60%	0.40%	5.40%
EIF3F	0.50%	1.00%	0.60%	0.00%	0.00%	0.00%	0.90%	4.50%	0.20%	0.30%	1.00%	0.40%	0.00%	0.50%	0.00%	0.60%	1.20%	0.20%	0.00%	0.90%

Supplementary Table 2: TCGA pan-cancer genomic interrogation of somatic mutations in the 84 DUBs

STUDY_ABBREVIATION	AML (TCGA)	Bladder (TCGA)	Breast (TCGA)	ccRCC (TCGA)	Cervical (TCGA)	Colorectal (TCGA)	GBM (TCGA)	Glioma (TCGA)	Head & neck (TCGA)	Liver (TCGA)	Lung adeno (TCGA)	Lung squ (TCGA)	Melanoma (TCGA)	Ovarian (TCGA)	pRCC (TCGA)	Prostate (TCGA)	Sarcoma (TCGA)	Stomach (TCGA)	Thyroid (TCGA)	Uterine (TCGA)
**samples**	200	413	1105	538	309	633	604	530	530	442	522	504	479	603	293	499	265	478	516	548
**UCHL1**	0.00%	0.80%	0.00%	0.00%	0.50%	0.90%	0.00%	0.00%	0.00%	0.30%	0.40%	0.00%	1.90%	0.00%	0.00%	0.00%	0.00%	0.80%	0.00%	1.60%
**UCHL3**	0.00%	0.80%	0.20%	0.20%	0.00%	0.90%	0.00%	0.00%	0.20%	0.50%	0.00%	0.60%	0.50%	0.00%	0.00%	0.00%	0.00%	1.00%	0.00%	0.00%
**BAP1**	0.00%	3.80%	0.80%	8.90%	2.10%	0.90%	0.30%	0.00%	0.60%	5.60%	1.30%	0.60%	1.90%	0.60%	5.00%	0.00%	0.00%	2.80%	0.20%	4.00%
**UCHL5**	0.00%	0.80%	0.20%	0.00%	0.00%	0.40%	0.00%	0.00%	0.60%	0.50%	0.90%	2.30%	0.30%	0.30%	0.00%	0.00%	0.00%	0.30%	0.00%	1.20%
**USP1**	0.00%	0.80%	0.60%	0.20%	2.10%	0.40%	0.00%	0.00%	1.00%	1.10%	3.00%	0.60%	1.10%	0.30%	0.00%	0.00%	0.00%	1.30%	0.00%	2.00%
**USP2**	0.00%	0.80%	0.20%	0.90%	0.00%	2.20%	0.30%	0.00%	0.00%	0.50%	0.90%	0.60%	2.40%	0.30%	0.00%	0.20%	0.00%	2.30%	0.00%	3.20%
**USP3**	0.00%	0.80%	0.10%	0.40%	0.00%	2.70%	0.00%	0.00%	0.80%	0.50%	0.40%	0.60%	0.30%	0.00%	0.70%	0.20%	0.00%	1.30%	0.00%	2.00%
**USP4**	0.00%	3.10%	0.60%	0.20%	2.10%	1.30%	0.00%	0.00%	1.20%	0.80%	0.40%	0.60%	1.60%	0.90%	0.70%	0.00%	0.40%	1.50%	0.00%	2.40%
**USP5**	0.00%	0.80%	0.60%	0.40%	0.00%	1.80%	0.00%	0.00%	1.00%	1.10%	1.30%	1.10%	1.90%	0.30%	0.70%	0.20%	0.00%	1.50%	0.20%	4.40%
**USP6**	0.50%	1.50%	0.40%	0.40%	2.10%	2.20%	1.70%	0.30%	2.00%	1.10%	2.20%	4.50%	8.20%	0.00%	0.70%	0.80%	1.60%	3.00%	0.20%	4.80%
**USP7**	0.00%	2.30%	0.60%	0.40%	2.10%	3.60%	0.30%	0.00%	1.40%	1.60%	3.90%	2.80%	4.30%	0.90%	0.00%	0.40%	0.40%	3.30%	0.00%	2.40%
**USP8**	0.00%	3.80%	0.60%	0.20%	2.60%	1.80%	0.00%	0.00%	2.10%	1.10%	1.70%	2.30%	2.70%	0.30%	0.00%	0.40%	0.40%	1.80%	0.00%	3.60%
**USP9X**	0.50%	3.10%	1.70%	2.00%	4.10%	3.10%	0.70%	1.40%	4.70%	1.90%	3.50%	3.40%	5.20%	0.30%	0.70%	1.00%	0.00%	4.80%	1.00%	10.50%
**USP10**	0.50%	0.80%	0.20%	0.20%	0.50%	2.70%	0.00%	0.00%	1.00%	0.00%	0.40%	0.60%	2.70%	0.00%	0.40%	0.20%	0.40%	2.00%	0.20%	4.00%
**USP11**	0.50%	0.00%	0.40%	0.70%	2.60%	3.10%	1.00%	1.00%	1.40%	0.30%	2.20%	3.40%	2.70%	0.90%	0.40%	0.00%	0.00%	2.30%	0.00%	2.80%
**USP12**	0.00%	0.80%	0.60%	0.20%	0.50%	0.90%	0.30%	0.00%	0.40%	0.30%	0.40%	0.00%	0.30%	0.00%	0.70%	0.60%	0.00%	1.00%	0.00%	2.80%
**USP13**	0.00%	3.10%	0.70%	0.00%	0.00%	2.20%	0.00%	0.00%	1.20%	0.30%	0.40%	5.60%	1.40%	0.00%	0.40%	0.00%	0.80%	3.00%	0.00%	5.20%
**USP14**	0.00%	0.80%	0.10%	0.00%	0.00%	0.40%	0.00%	0.30%	0.20%	0.30%	1.30%	0.00%	0.30%	0.30%	0.40%	0.20%	0.00%	2.00%	0.00%	2.00%
**USP15**	0.00%	0.80%	0.70%	1.30%	1.50%	2.70%	0.70%	0.00%	1.80%	1.10%	1.70%	2.30%	2.70%	0.60%	0.40%	1.00%	0.00%	1.80%	0.00%	2.40%
**USP16**	0.00%	0.80%	0.10%	0.20%	0.00%	2.20%	0.30%	0.00%	0.80%	0.30%	0.40%	1.10%	1.90%	0.00%	0.00%	0.20%	0.00%	2.00%	0.00%	5.60%
**USP17L2**	0.00%	0.00%	0.10%	0.20%	2.10%	3.60%	0.00%	0.00%	1.80%	0.00%	1.30%	1.10%	6.30%	0.30%	0.00%	0.00%	0.40%	1.30%	0.00%	0.40%
**USP18**	0.00%	0.00%	0.00%	0.90%	0.50%	0.40%	0.00%	0.00%	0.20%	0.00%	0.40%	0.60%	0.30%	0.30%	0.00%	0.00%	0.40%	0.00%	0.00%	1.60%
**USP19**	0.00%	1.50%	0.20%	0.20%	2.10%	2.20%	0.00%	0.00%	1.80%	1.10%	1.70%	1.10%	5.40%	0.30%	1.10%	0.40%	0.00%	4.10%	0.00%	4.00%
**USP20**	0.00%	3.10%	0.20%	0.20%	1.50%	1.30%	0.70%	0.70%	0.20%	1.60%	0.00%	1.10%	1.60%	0.30%	1.40%	0.40%	0.40%	2.30%	0.20%	3.60%
**USP21**	0.00%	1.50%	0.40%	0.20%	1.00%	1.80%	0.00%	0.00%	0.60%	0.30%	0.90%	2.30%	2.70%	0.60%	0.00%	0.00%	0.00%	3.30%	0.00%	4.40%
**USP22**	0.00%	0.00%	0.20%	0.20%	0.00%	0.90%	0.30%	0.00%	0.80%	0.80%	0.40%	0.00%	0.50%	0.30%	0.00%	0.40%	0.00%	2.00%	0.00%	3.20%
**USP24**	0.50%	3.80%	0.80%	0.90%	2.60%	2.20%	0.00%	0.30%	0.60%	1.90%	2.60%	3.40%	3.80%	1.60%	0.00%	0.80%	1.20%	4.60%	0.00%	6.90%
**USP25**	0.00%	1.50%	0.60%	0.40%	0.50%	2.20%	0.30%	1.00%	0.60%	0.50%	1.70%	3.40%	3.00%	0.30%	1.80%	0.00%	0.40%	1.80%	0.00%	7.30%
**USP26**	0.00%	1.50%	0.50%	0.70%	3.60%	3.10%	0.70%	0.30%	1.40%	0.80%	4.80%	2.30%	8.20%	0.30%	0.00%	0.40%	0.00%	5.60%	0.00%	5.20%
**USP27X**	0.00%	0.00%	0.00%	0.00%	0.00%	0.00%	0.00%	0.00%	0.20%	0.50%	0.00%	0.00%	0.00%	0.00%	0.00%	0.00%	0.40%	0.30%	0.00%	2.40%
**USP28**	0.00%	3.10%	0.40%	0.70%	2.10%	4.90%	1.00%	0.00%	1.60%	0.80%	3.00%	2.30%	4.60%	0.30%	1.10%	0.80%	0.40%	4.30%	0.20%	4.00%
**USP29**	0.00%	2.30%	0.60%	0.70%	1.50%	3.60%	0.70%	0.00%	3.30%	0.80%	6.10%	9.60%	13.90%	0.60%	0.70%	0.60%	0.00%	2.50%	0.00%	4.40%
**USP30**	0.00%	0.00%	0.10%	2.00%	0.50%	0.40%	0.00%	0.30%	0.60%	0.30%	0.00%	1.70%	1.60%	0.00%	0.70%	0.40%	0.40%	2.30%	0.00%	4.00%
**USP31**	0.00%	2.30%	0.50%	0.40%	3.10%	4.90%	0.00%	0.70%	2.00%	1.30%	2.20%	1.10%	5.40%	0.60%	0.00%	1.00%	0.40%	4.80%	0.00%	5.20%
**USP32**	0.00%	3.10%	0.80%	0.40%	2.60%	3.60%	0.30%	0.00%	1.40%	0.50%	0.40%	2.80%	3.30%	0.00%	0.70%	0.40%	0.00%	3.00%	0.00%	6.00%
**USP33**	0.00%	2.30%	0.50%	0.40%	1.50%	3.60%	0.00%	0.00%	2.10%	0.00%	2.60%	3.40%	3.00%	0.30%	0.00%	0.40%	0.00%	1.50%	0.00%	3.60%
**USP34**	0.50%	5.40%	2.30%	1.80%	4.60%	4.00%	0.30%	0.00%	4.90%	4.00%	6.10%	10.70%	5.40%	1.60%	2.10%	0.60%	0.80%	7.30%	0.20%	8.10%
**USP35**	0.00%	0.80%	0.40%	0.70%	1.00%	0.40%	1.00%	0.30%	1.80%	0.50%	0.90%	1.70%	1.10%	0.30%	0.40%	0.40%	0.40%	3.50%	0.00%	4.00%
**USP36**	0.00%	0.80%	0.80%	1.30%	1.00%	2.20%	0.00%	0.00%	1.60%	0.80%	0.40%	1.70%	2.40%	0.60%	0.70%	0.40%	0.40%	3.30%	0.00%	6.00%
**USP37**	0.00%	0.80%	0.50%	0.70%	2.10%	0.40%	0.00%	0.70%	0.40%	1.10%	0.40%	2.80%	3.00%	0.30%	0.70%	0.60%	0.40%	2.30%	0.20%	4.00%
**USP38**	0.00%	3.80%	0.20%	0.00%	1.00%	1.30%	0.00%	0.00%	1.00%	1.90%	1.30%	0.60%	0.80%	0.30%	0.40%	0.20%	0.40%	2.50%	0.00%	4.00%
**USP39**	0.00%	1.50%	0.60%	1.80%	0.00%	0.90%	0.30%	0.00%	0.00%	0.00%	0.00%	0.60%	0.80%	0.00%	0.40%	0.00%	0.00%	1.00%	0.00%	2.40%
**USP40**	0.00%	1.50%	0.50%	0.90%	1.50%	2.20%	0.00%	0.30%	1.00%	1.30%	1.30%	1.70%	3.30%	0.60%	0.40%	0.20%	0.00%	3.00%	0.20%	3.60%
**USP41**	0.00%	0.00%	0.20%	0.20%	0.00%	0.00%	0.30%	0.00%	0.20%	0.00%	0.40%	0.00%	0.50%	0.00%	0.00%	0.00%	0.00%	0.00%	0.20%	1.60%
**USP42**	0.00%	1.50%	0.50%	0.20%	1.50%	0.90%	1.00%	0.00%	0.80%	0.50%	1.30%	2.30%	1.90%	0.00%	0.70%	0.20%	0.40%	3.50%	0.00%	3.60%
**USP43**	0.00%	2.30%	0.40%	0.70%	4.10%	1.30%	0.00%	0.30%	0.20%	0.50%	0.40%	0.60%	4.90%	0.00%	0.40%	0.00%	0.00%	2.30%	0.00%	3.60%
**USP44**	0.00%	0.80%	0.00%	0.40%	0.50%	4.50%	0.00%	0.70%	0.80%	1.10%	1.30%	0.00%	3.50%	0.30%	0.40%	0.20%	0.40%	2.30%	0.00%	4.00%
**USP45**	0.00%	0.00%	0.70%	0.40%	0.50%	2.20%	0.30%	0.00%	0.60%	0.50%	0.00%	0.60%	1.10%	0.30%	0.40%	0.40%	0.40%	1.80%	0.00%	4.40%
**USP46**	0.00%	0.00%	0.10%	0.00%	0.50%	0.90%	0.00%	0.00%	0.80%	0.00%	1.30%	1.70%	0.00%	0.60%	0.40%	0.40%	0.40%	0.80%	0.20%	2.40%
**USP47**	0.00%	3.10%	0.50%	0.20%	1.50%	1.80%	0.30%	0.00%	1.00%	1.60%	1.30%	2.30%	1.90%	0.00%	0.40%	0.20%	0.80%	1.80%	0.00%	5.20%
**USP48**	0.50%	3.00%	0.00%	1.00%	3.10%	2.70%	0.00%	0.00%	1.00%	2.00%	1.70%	1.00%	1.00%	1.00%	0.40%	1.00%	0.80%	2.00%	0.20%	5.20%
**USP49**	0.00%	0.80%	0.30%	0.20%	1.00%	0.40%	0.70%	0.00%	0.80%	0.80%	2.20%	1.10%	1.60%	0.90%	0.00%	0.00%	0.00%	2.50%	0.20%	1.60%
**USP50**	0.00%	0.00%	0.10%	0.40%	0.50%	1.30%	0.00%	0.00%	0.00%	0.80%	0.00%	1.10%	1.10%	0.00%	0.00%	0.00%	0.00%	0.30%	0.00%	0.40%
**USP51**	0.00%	0.00%	0.30%	1.10%	2.60%	1.30%	0.70%	0.70%	1.00%	0.00%	3.00%	1.10%	0.50%	0.30%	0.00%	0.00%	0.00%	3.00%	0.20%	2.00%
**USP52**	0.00%	6.20%	0.50%	0.70%	1.50%	0.40%	0.30%	0.00%	2.00%	1.30%	1.30%	2.80%	3.30%	0.30%	1.10%	0.80%	0.40%	4.60%	0.00%	3.20%
**USP53**	0.00%	0.80%	0.50%	0.20%	2.10%	2.70%	0.30%	0.70%	1.40%	1.10%	0.90%	0.60%	2.40%	0.30%	0.40%	0.40%	0.00%	2.00%	0.00%	3.60%
**USP54**	0.00%	0.80%	0.50%	0.40%	1.50%	2.70%	0.00%	0.00%	2.00%	1.30%	1.70%	4.00%	4.10%	0.60%	0.40%	0.20%	0.00%	3.50%	0.00%	6.00%
**CYLD**	0.00%	0.00%	0.20%	0.40%	1.00%	1.80%	0.30%	0.00%	2.90%	1.10%	3.00%	4.50%	2.20%	0.00%	0.00%	0.20%	0.00%	3.80%	0.00%	5.20%
**CEZANNE**	0.00%	1.50%	0.80%	0.40%	0.50%	2.20%	0.00%	0.30%	1.40%	0.80%	0.90%	2.30%	3.50%	0.00%	0.40%	0.00%	0.40%	2.50%	0.00%	4.40%
**CEZANNE2**	0.00%	4.60%	0.80%	0.20%	0.50%	0.90%	0.30%	0.30%	0.80%	0.30%	2.20%	1.70%	3.50%	0.00%	0.00%	0.40%	0.00%	2.80%	0.00%	3.20%
**TRABID**	0.00%	2.30%	0.50%	0.90%	0.50%	0.90%	0.00%	0.00%	0.80%	1.10%	0.00%	1.70%	0.50%	0.60%	0.00%	0.20%	0.00%	2.80%	0.00%	2.40%
**OTUD4**	0.00%	6.90%	0.80%	0.70%	1.00%	2.70%	0.70%	0.00%	0.80%	0.00%	1.70%	2.30%	3.30%	0.90%	0.70%	1.40%	0.00%	3.80%	1.20%	5.20%
**ALG13**	0.50%	0.80%	0.50%	0.90%	2.60%	0.00%	0.00%	0.30%	0.80%	0.30%	2.60%	3.40%	3.30%	0.00%	0.00%	0.20%	0.40%	2.00%	0.50%	7.30%
**OTUD3**	0.00%	0.00%	0.10%	0.40%	1.00%	1.80%	0.00%	0.30%	0.00%	0.30%	0.00%	0.00%	0.50%	0.00%	0.70%	0.40%	0.00%	0.50%	0.00%	1.20%
**OTUD1**	0.00%	0.00%	0.10%	0.00%	0.50%	0.00%	0.00%	0.00%	0.00%	0.30%	0.00%	0.00%	0.00%	0.00%	0.40%	0.00%	0.00%	0.00%	0.00%	0.00%
**OTUD6A**	0.00%	2.30%	0.30%	0.20%	0.50%	0.90%	0.30%	0.00%	0.40%	0.00%	1.30%	1.10%	2.40%	0.00%	0.00%	0.00%	0.00%	0.50%	0.20%	1.20%
**OTUD2/YOD1**	0.00%	0.80%	0.10%	0.20%	0.00%	0.40%	0.70%	0.00%	0.80%	0.00%	0.40%	0.00%	0.30%	0.60%	0.40%	0.00%	0.40%	0.30%	0.00%	0.80%
**OTUD6B**	0.00%	0.00%	0.00%	0.00%	0.00%	1.30%	0.00%	0.00%	0.00%	0.00%	0.00%	0.60%	0.00%	0.00%	0.00%	0.20%	0.40%	0.50%	0.00%	0.00%
**OTUB1**	0.00%	0.00%	0.10%	0.00%	0.00%	0.40%	0.00%	0.30%	0.20%	0.80%	0.40%	0.00%	0.50%	0.00%	0.40%	0.20%	0.40%	0.80%	0.20%	1.20%
**OTUB2**	0.00%	0.80%	0.00%	0.20%	0.00%	0.40%	0.00%	0.00%	0.60%	0.00%	0.40%	0.00%	0.30%	0.00%	0.00%	0.00%	0.00%	0.30%	0.00%	0.80%
**OTUD5**	0.00%	0.80%	0.00%	0.00%	2.10%	0.90%	0.30%	0.00%	0.40%	0.30%	1.30%	0.60%	0.80%	0.00%	0.00%	0.20%	0.00%	1.00%	0.20%	2.40%
**ATXN3**	0.00%	2.30%	0.30%	0.40%	0.50%	0.40%	0.00%	0.00%	1.20%	0.30%	0.00%	2.80%	0.30%	0.00%	0.40%	0.20%	0.40%	1.30%	0.00%	2.00%
**JOSD1**	0.00%	0.80%	0.00%	0.00%	0.50%	0.40%	0.00%	0.00%	0.00%	0.00%	0.00%	0.00%	0.00%	0.30%	0.00%	0.20%	0.40%	0.50%	0.00%	0.80%
**JOSD2**	0.00%	0.80%	0.00%	0.20%	0.00%	0.00%	0.30%	0.00%	0.20%	0.00%	0.00%	0.00%	0.50%	0.00%	0.00%	0.00%	0.00%	0.30%	0.00%	0.80%
**TAF1D**	0.00%	0.00%	0.00%	0.20%	0.00%	0.40%	0.00%	0.00%	0.20%	1.10%	0.90%	0.00%	0.00%	0.00%	0.00%	0.80%	0.00%	1.80%	0.00%	1.20%
**MCPIP1**	0.00%	1.50%	0.50%	0.20%	2.60%	0.90%	0.30%	0.00%	0.20%	1.10%	0.90%	1.10%	1.60%	0.60%	0.70%	0.20%	0.40%	3.50%	0.00%	3.20%
**MCPIP2**	0.00%	1.50%	0.90%	0.40%	1.50%	1.80%	0.70%	0.70%	0.60%	0.50%	3.50%	1.70%	3.00%	0.00%	0.00%	0.00%	0.00%	2.00%	0.00%	5.60%
**MCPIP3**	0.00%	0.80%	0.60%	0.00%	1.50%	4.00%	0.00%	0.00%	1.20%	1.30%	1.70%	2.30%	2.20%	0.00%	0.40%	0.20%	0.40%	1.80%	0.20%	4.00%
**MCPIP4**	0.00%	0.00%	0.00%	0.20%	0.00%	0.90%	0.00%	0.00%	0.00%	0.80%	0.00%	0.00%	1.10%	0.00%	0.00%	0.40%	0.00%	1.00%	0.00%	2.40%
**POH1**	0.00%	0.00%	0.10%	0.00%	0.00%	0.90%	0.00%	0.00%	0.20%	0.30%	0.40%	1.70%	0.00%	0.00%	0.00%	0.20%	0.00%	0.30%	0.00%	0.80%
**CSN5**	0.00%	0.00%	0.00%	0.20%	1.00%	0.40%	0.00%	0.00%	0.40%	0.30%	0.90%	1.70%	1.10%	0.00%	0.40%	0.00%	0.00%	0.80%	0.00%	2.00%
**CSN6**	0.00%	0.00%	0.30%	0.20%	0.00%	0.90%	0.00%	0.00%	0.20%	0.30%	0.00%	0.00%	0.50%	0.00%	0.40%	0.20%	0.00%	0.80%	0.00%	2.40%
**AMSH**	0.00%	0.80%	0.30%	0.40%	0.00%	0.90%	0.00%	0.00%	0.40%	0.00%	0.00%	1.10%	0.80%	0.30%	0.00%	0.20%	0.00%	0.80%	0.00%	2.40%
**BRCC3**	0.50%	0.00%	0.20%	0.40%	1.50%	0.00%	0.30%	0.00%	0.20%	0.30%	0.90%	0.60%	0.50%	0.30%	0.40%	0.20%	0.00%	0.50%	0.00%	3.20%
**MYSM1**	0.00%	1.50%	0.30%	0.20%	1.00%	0.90%	0.70%	0.00%	0.80%	0.50%	0.90%	1.10%	3.00%	0.00%	0.70%	0.20%	0.00%	1.50%	0.00%	3.60%
**MPND**	0.50%	1.50%	0.10%	0.20%	0.00%	0.40%	0.00%	0.00%	0.40%	0.30%	0.00%	0.60%	0.30%	0.30%	0.70%	0.00%	0.00%	1.00%	0.00%	2.00%
**PSMD7**	0.00%	0.00%	0.10%	0.20%	0.00%	0.40%	0.00%	0.00%	0.00%	0.00%	0.90%	1.70%	0.30%	0.00%	0.40%	0.20%	0.00%	1.30%	0.00%	1.60%
**PRPF8**	1.00%	1.50%	1.20%	2.40%	2.60%	4.50%	0.70%	0.30%	2.00%	2.10%	0.40%	3.40%	5.20%	0.30%	1.40%	1.00%	0.80%	4.30%	0.50%	8.50%
**EIF3H**	0.00%	0.00%	0.10%	0.00%	1.00%	0.40%	0.30%	0.00%	0.20%	0.30%	0.00%	1.10%	0.80%	0.00%	0.00%	0.00%	0.00%	1.00%	0.00%	1.20%
**EIF3F**	0.00%	0.80%	0.10%	0.20%	0.00%	1.30%	0.00%	0.00%	0.00%	0.30%	0.00%	1.10%	0.30%	0.00%	0.00%	0.20%	0.00%	0.50%	0.20%	0.80%

Supplementary Table 3: The ovarian cancer patient cohort for USP13 and MCL1 IHC

Number	Age	Pathological type	Clinical Stage
1	73	Serous ovarian cancer	Ia
2	64	Mucinous ovarian cancer	IIb
3	61	Serous ovarian cancer	IIIc
4	60	Serous ovarian cancer	IIIb
5	70	Endometrioid ovarian cancer	IIIc
6	61	Ovarian cancer	IIIc
7	59	Endometrioid ovarian cancer	Ia
8	52	Mucinous ovarian cancer	Ia
9	62	Serous ovarian cancer	IV
10	55	Serous ovarian cancer	IIIc
11	55	Serous ovarian cancer	IV
12	58	Ovarian cancer	III
13	42	Serous ovarian cancer	IIIc
14	64	Serous ovarian cancer	IIIa
15	77	Ovarian cancer	IIc
16	75	Serous ovarian cancer	IIb
17	85	Ovarian cancer	III
18	45	Serous ovarian cancer	IV
19	60	Serous ovarian cancer	IIIc
20	52	Ovarian cancer	IIIc
21	52	Serous ovarian cancer	IIIc
22	54	Serous ovarian cancer	IV
23	45	Serous ovarian cancer	IIIc
24	54	Serous ovarian cancer	IV
25	72	Serous ovarian cancer	IIIc
26	73	Serous ovarian cancer	IIc
27	67	Serous ovarian cancer	IV
28	68	Serous ovarian cancer	IIIc
29	61	Ovarian cancer	Ia
30	67	Serous ovarian cancer	IIIc
31	65	Ovarian cancer	IIIc
32	67	Endometrioid ovarian cancer	IIIc
33	66	Ovarian cancer	IIc
34	42	Serous ovarian cancer	IIIc
35	53	Serous ovarian cancer	IIIa
36	53	Ovarian cancer	IIIa
37	65	Clear-cell ovarian cancer	Ia
38	52	Clear-cell ovarian cancer	Ic
39	48	Serous ovarian cancer	IIIc
40	68	Serous ovarian cancer	IIIc
41	67	Serous ovarian cancer	Ia
42	47	Ovarian cancer	Ic
43	29	Ovarian cancer	IIIa
44	64	Ovarian cancer	IV
45	39	Ovarian cancer	Ia
46	60	Ovarian cancer	IIIc
47	46	Serous ovarian cancer	IIIc
48	50	Serous ovarian cancer	IIIc
49	49	Serous ovarian cancer	IV
50	59	Serous ovarian cancer	IIIc

Supplementary Table 4: The lung cancer patient cohort for USP13 and MCL1 IHC

Number	Sex	Age	Pathological type	Clinical stage
RRsLug0906A0726	female	66	lung adenocarcinoma	Ⅰ
RRsLug0906A0722	female	68	lung adenocarcinoma	Ⅰ
E05A0759	female	69	lung adenocarcinoma	Ⅰ
RRsLug0801A0450	female	58	lung adenocarcinoma	Ⅰ - Ⅱ
E05A0796	female	63	lung adenocarcinoma	Ⅰ - Ⅱ
CRsLug0612A0232	female	71	lung adenocarcinoma	Ⅱ
CRsLug0612A0238	male	51	lung adenocarcinoma	Ⅱ
CRsLug0702A0260	female	56	lung adenocarcinoma	Ⅱ
CRsLug0702A0264	female	30	lung adenocarcinoma	Ⅱ
CRsLug0706A0369	male	42	lung adenocarcinoma	Ⅱ
CRsLug0707A0372	male	63	lung adenocarcinoma	Ⅱ
CRsLug0709A0392	female	57	lung adenocarcinoma	Ⅱ
CRsLug0709A0399	female	51	lung adenocarcinoma	Ⅱ
CRsLug0711A0441	female	60	lung adenocarcinoma	Ⅱ
CRsLug0804A0507	female	61	lung adenocarcinoma	Ⅱ
CRsLug0806A0539	female	50	lung adenocarcinoma	Ⅱ
CRsLug0806A0542	male	70	lung adenocarcinoma	Ⅱ
CRsLug0812A0627	male	59	lung adenocarcinoma	Ⅱ
CRsLug0812A0629	male	40	lung adenocarcinoma	Ⅱ
CRsLug0902A0648	female	62	lung adenocarcinoma	Ⅱ
CRsLug0612A0230	female	52	lung adenocarcinoma	Ⅱ - Ⅲ
CRsLug0711A0433	female	63	lung adenocarcinoma	Ⅱ - Ⅲ
CRsLug0711A0438	male	60	lung adenocarcinoma	Ⅱ - Ⅲ
CRsLug0806A0559	female		lung adenocarcinoma	Ⅱ - Ⅲ
NRsLug0312A0002	female	37	lung adenocarcinoma	Ⅲ
CRsLug0512A0149	male	67	lung adenocarcinoma	Ⅲ
CRsLug0709A0388	female	63	lung adenocarcinoma	Ⅲ
CRsLug0812A0623	male	67	lung adenocarcinoma	Ⅲ
CRsLug0902A0636	male	67	lung adenocarcinoma	Ⅲ
CRsLug0903A0657	male	62	lung adenocarcinoma	Ⅲ
