# Supplementary material for: Deubiquitinase USP13 dictates MCL1 stability and sensitivity to BH3 mimetic inhibitors
Source: Nat Commun. 2018 Jan 15;9:215. doi: 10.1038/s41467-017-02693-9 (PMC5768685; doi:10.1038/s41467-017-02693-9)
Supplement: Supplementary file 3 — Description of Additional Supplementary Files [file 41467_2017_2693_MOESM3_ESM.pdf]

Description of Additional Supplementary Files

File Name: Supplementary Data 1
Description: The siRNA library of 84 DUBs

File Name: Supplementary Data 2
Description: Screening of 180 small molecule inhibitors targeting wide-ranging signalling pathways using MCL1-depleted SW-1573 cells.
